# Broken colinearity of the amphioxus *Hox* cluster

**DOI:** 10.1186/2041-9139-3-28

**Published:** 2012-12-03

**Authors:** Juan Pascual-Anaya, Noritaka Adachi, Susana Álvarez, Shigeru Kuratani, Salvatore D’Aniello, Jordi Garcia-Fernàndez

**Affiliations:** 1 Departament de Genètica and Institut de Biomedicina (IBUB), University of Barcelona, Av. Diagonal, 643, Barcelona, 08028, Spain; 2 Laboratory for Evolutionary Morphology, RIKEN Center for Developmental Biology, 2-2-3 Minatojima-minamimachi, Kobe, Hyogo, 650-0047, Japan; 3 Department of Organic Chemistry, Universidade de Vigo, Vigo, Pontevedra, 36310, Spain; 4 Cellular and Developmental Biology, Stazione Zoologica Anton Dohrn, Villa Comunale, Naples, 80121, Italy

**Keywords:** *Hox* gene regulation, *Hox* cluster evolution, Amphioxus, *Hox* colinearity, Retinoic acid

## Abstract

**Background:**

In most eumetazoans studied so far, *Hox* genes determine the identity of structures along the main body axis. They are usually linked in genomic clusters and, in the case of the vertebrate embryo, are expressed with spatial and temporal colinearity. Outside vertebrates, temporal colinearity has been reported in the cephalochordate amphioxus (the least derived living relative of the chordate ancestor) but only for anterior and central genes, namely *Hox1* to *Hox4* and *Hox6*. However, most of the *Hox* gene expression patterns in amphioxus have not been reported. To gain global insights into the evolution of *Hox* clusters in chordates, we investigated a more extended expression profile of amphioxus *Hox* genes.

**Results:**

Here we report an extended expression profile of the European amphioxus *Branchiostoma lanceolatum Hox* genes and describe that all *Hox* genes, except *Hox13*, are expressed during development. Interestingly, we report the breaking of both spatial and temporal colinearity for at least *Hox6* and *Hox14*, which thus have escaped from the classical *Hox* code concept. We show a previously unidentified *Hox6* expression pattern and a faint expression for posterior *Hox* genes in structures such as the posterior mesoderm, notochord, and hindgut. Unexpectedly, we found that amphioxus *Hox14* had the most divergent expression pattern. This gene is expressed in the anterior cerebral vesicle and pharyngeal endoderm. Amphioxus *Hox14* expression represents the first report of *Hox* gene expression in the most anterior part of the central nervous system. Nevertheless, despite these divergent expression patterns, amphioxus *Hox6* and *Hox14* seem to be still regulated by retinoic acid.

**Conclusions:**

Escape from colinearity by *Hox* genes is not unusual in either vertebrates or amphioxus and we suggest that those genes escaping from it are probably associated with the patterning of lineage-specific morphological traits, requiring the loss of those developmental constraints that kept them colinear.

## Background

**Figure 1:**
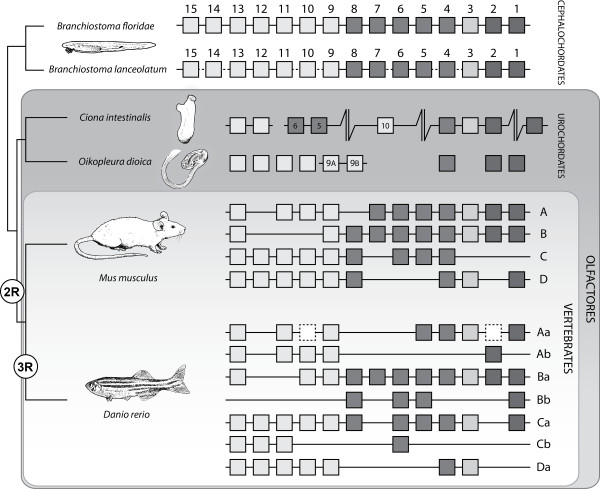
**Phylogenetic positions of cephalochordates,urochordates, and vertebrates,showing their *Hox *contents.** The cephalochordate amphioxus represents the basal branch of chordates and possesses a single *Hox* cluster of 15 genes in both *B*. *floridae* and *B*. *lanceolatum*, although the whole genomic sequence of the latter has not been reported yet (indicated by dashed lines in the corresponding regions). Urochordates, the sister group of vertebrates, possess a disintegrated *Hox* cluster at different levels. Whereas the ascidian *Ciona intestinalis* has a highly disintegrated cluster with several reorganizations, the larvacean *Oikopleura dioica* has a completely atomized cluster where only two *Hox9* genes remain linked, probably arising from an independent duplication. Vertebrates have multiple clusters, such as four in the mouse caused by two rounds of genome duplication (2R), or the seven clusters of zebrafish after a third teleost-specific round of genome duplication (3R). Squares represent *Hox* genes, with the same gray-scale colors indicating different paralogous groups (PG1 and PG2, PG3, central PG4-8, and posterior PG9-13/15). White boxes with dashed outlines represent pseudogenes.

*Hox* genes code for a subfamily of homeodomain-containing transcription factors and have been found in all eumetazoans studied so far. *Hox* genes are responsible for giving the identity to morphological structures along the anterior-posterior (A-P) axis in most bilaterian animals [1-4]. Generally, these genes lie in the same genomic region and form gene clusters, usually one in invertebrates, and multiple clusters in vertebrates because of multiple rounds of genome duplication that took place at their origin (Figure 1) [5,6]. In most groups of animals, the position of *Hox* genes within any cluster corresponds with their mode of expression: genes placed more toward the 3′ end are expressed and pattern more anterior structures than do genes placed at the 5^′^ end. As a result, *Hox* genes are expressed along the A-P axis in a nested manner with more rostral limits for 3^′^ than for 5^′^ genes. This phenomenon is called spatial colinearity [7]. Moreover, in the case of vertebrates, the 3^′^ genes are expressed in earlier stages of the developing embryo than are 5^′^ genes in what is known as temporal colinearity [8,9]. The different combinations of *Hox* genes expressed in different structures along the A-P axis constitute what is called the *Hox* code [10]. It is believed that changes in the patterns of *Hox* expression are somehow responsible for the appearance of some vertebrate innovations, such as the elaboration of the segmentation of the hindbrain [11].

Chordates include the group olfactores (vertebrates and urochordates) and the cephalochordates [12] (Figure 1). However, urochordates, as a reflection of their highly reorganized genome and extensive gene losses, do not retain the typical clustered organization of *Hox* genes with only some genes linked, as in the ascidian *Ciona intestinalis*[13,14] (Figure 1), or as an atomized cluster, as is the case of the larvacean *Oikopleura dioica*[15] (Figure 1). Nonetheless, the cephalochordate amphioxus, representing the most basal branch of chordates, has a rather prototypical genome [16] and possesses a single cluster of 15 *Hox* genes, where all of them are transcribed in the same orientation, as in vertebrates [17]. Thus, amphioxus represents the best model to compare with vertebrates for illuminating the basal condition of chordates for both *Hox* content and regulation. However, the expression of amphioxus *Hox* genes is scarcely reported and studies have focused mainly on the anterior ones. The genes of the Floridian amphioxus *Branchiostoma floridae Hox14* and *Hox6* have been reported to be expressed during development in a colinear manner in the central nervous system (CNS), highlighting that, although it is not morphologically segmented as in vertebrates, the CNS in both groups to some extent conserves the same nested *Hox* system [18]. In addition, the expression patterns of *Hox1*, *Hox3*, and *Hox4* have been reported in the epidermis and have been associated with the determination of different sensory neurons along the A-P axis [19]. Moreover, *Hox1* mRNA is expressed in the middle part of the gut [20].

The acidic form of vitamin A, retinoic acid (RA), has an important role in the regionalization of morphological structures along the A-P axis of vertebrates, acting as the main posteriorizing factor during neural determination [21]. Its function is carried out in a gradient-dependent manner, with higher concentrations in posterior parts [22]. In the case of amphioxus, an excess of RA during development also causes changes of anterior into posterior identities, and the mouth and gill slits do not form. Conversely, treatments with RA antagonists result in a caudal extension of the pharynx [23,24]. These effects caused by altered concentrations of RA during development are equivalent to those observed in vertebrate embryos and highlight that determination mechanisms of the structures along the A-P axis are somehow conserved in chordates. RA carries out its function by binding heterodimers of the RA receptor (RAR) and retinoid X receptor (RXR), which regulate the transcription of their target genes by binding to RA response elements (RAREs) in the regulatory regions of the genome. RAREs consist of two direct repeats (DRs) separated by a variable number of nucleotides. In the case of RAR/RXR heterodimers, they have been shown to bind DRs separated by one (DR1), two (DR2), or five (DR5) nucleotides [25]. RA has an important role in controlling *Hox* genes [26,27] via RAR/RXR binding to RAREs [28-30]. Besides, the anterior *Hox* genes in amphioxus are regulated by RA [18-20], and morpholino knock-down of *Hox1* produces the same phenotype as treatment with an RA antagonist, indicating that *Hox1* mediates the function of RA to establish the posterior limit of the pharynx [20]. Therefore, the regulation of anterior *Hox* genes by RA seems to be conserved between vertebrates and amphioxus, as suggested by heterologous reporter assays using regulatory regions of amphioxus *Hox* genes in both the mouse and chicken [31,32]. Again, most of these studies were focused on the anterior part of the cluster; hence, a general scenario for *Hox* cluster regulatory evolution is not yet available.

In this study, we report previously undescribed expression patterns of *Hox* genes of the European amphioxus *Branchiostoma lanceolatum*, and, surprisingly, find that some of them are not expressed in a colinear manner either in space or time, thus breaking the paradigm of *Hox* colinearity in amphioxus. We identified a different expression for *B*. *lanceolatum Hox6* than that previously reported for the Floridian amphioxus [18,33] and detected *Hox14* expression in the pharyngeal endoderm at the level of the endostyle, the mid-hindgut, and notochord. Strikingly, it was detected in the cerebral vesicle, a part of the CNS where no *Hox* expression has been detected so far. Thus, this gene has escaped from the *Hox* coding pattern, as has been reported for vertebrate *Hox14* genes [34,35]. We then investigated the regulation of these genes by RA and found that RA regulated the expression of *Hox6* and the expressions of *Hox14* in the gut and notochord. It also affected *Hox14* expression in the cerebral vesicle, whereas the regulation of *Hox14* in the pharyngeal endoderm seemed to be RA independent. The presence of RAREs near these genes, conserved between both the Floridian and European amphioxus species, makes these genes colinearity-breakers but still likely targets of RA regulation.

## Methods

### Embryonic culture and treatment with RA and the RA antagonist BMS009

Sexually mature amphioxus adults (*B*. *lanceolatum*) were collected in Argelés-sur-Mer (France) during the summer of 2009. Spawning was induced in the laboratory by heat shock [36]. After fertilization, embryos were reared in filtered seawater at 17°C. Treatments with RA (in DMSO), the RA antagonist BMS009 (in DMSO), or equivalent amounts of DMSO (as control) were carried out at the late blastula stage at a final concentration of 1 × 10^-6^ M as described [23,24]. At the early neurula stage, embryos were transferred to untreated filtered seawater, washed a few times, and kept in normal conditions. The control DMSO treatment did not affect development. Embryos and larvae were fixed at frequent intervals with 4% paraformaldehyde overnight at 4°C in a buffer containing 0.1 M MOPS, 0.5 M NaCl, 2 mM MgSO_4_, 1 mM EGTA, pH 7.4 for *in situ* hybridization.

### Extension of the genomic region of *B*. *lanceolatum Hox11*

Because the coding sequences of *B*. *floridae* and *B*. *lanceolatum* are extremely conserved, we decided to use primers based on the *B*. *floridae Hox11* exon 2 to amplify the first intron of *B*. *lanceolatum's* one. Using a forward primer from the *B*. *lanceolatum Hox11* exon 1 (5^′^-ATGGACGGTTACTGGCTGC-3′, [37]) and a reverse primer designed on the *B*. *floridae Hox11* exon 2 sequence (5^′^-CTGCCTATCCGTGAGGTTG-3^′^, [38]), we amplified a band of approximately 2.5 Kbp using *B*. *lanceolatum* genomic DNA as a template. We cloned it into pGEM-T Easy Vector (Promega) and sequenced it. The sequence corresponded to the first exon, first intron, and second exon of *B*. *lanceolatum Hox11*. The genomic sequence with the new annotation has been uploaded to the NCBI GenBank Database ( http://www.ncbi.nlm.nih.gov/genbank/) under accession no. JX508623.

### cDNA cloning, whole-mount *in situ* hybridization (WISH), microscopy, and photography

**Figure 2:**
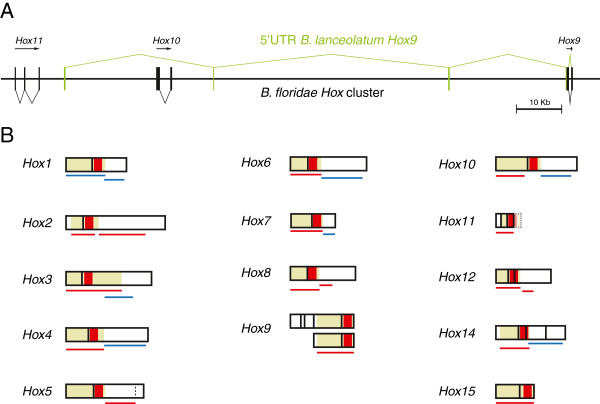
**5**^**′**^**-UTR of *B. lanceolatum Hox9 *andprobes used in this study.** (**A**) Scheme of the *B*. *floridae Hox* cluster with *Hox11*, *Hox10*, and *Hox9* represented. The *B*. *lanceolatum Hox9* transcripts consist of two different versions: a large one, with three 5′-UTR (in green) exons far upstream of the *Hox9* locus, with its splicing shown at the top. A shorter one, with a canonical 5′-UTR next to the first exon of *Hox9*, is shown with its splicing represented at the bottom. Black vertical lines represent exons of the different *Hox* genes. (**B**) The transcripts of all the *B*. *lanceolatum Hox* genes found to be expressed in this study. Color-coded boxes represent exons: beige, coding sequences; white, UTRs; and red, homeoboxes. The lines under the transcripts represent the probes used: red, a negative probe; blue, a positive probe. The third exon of *B*. *lanceolatum Hox11* has not yet been described, so it is represented with a dashed line and fainter colors.

A mix of embryo stages from gastrula to 2-day-old larvae of *B*. *lanceolatum* were fixed in RNAlater (Ambion) and total RNA was extracted using RNeasy Mini Kit (QIAGEN). The cDNA first strand was synthesized using Superscript III Reverse Transcriptase (Invitrogen) (1 h, 56°C). An embryo cDNA library was constructed using the CloneMiner Kit (pDNR222 vector; Invitrogen). Reverse transcription polymerase chain reaction (RT-PCR) amplification of the coding DNA sequence (CDS) and 5′- or 3′-rapid amplification of cDNA ends (RACE; Invitrogen) for all *B*. *lanceolatum Hox* genes (*Hox115*) were carried out with primers designed based on the sequences reported previously [37], except for *Hox11*, where we used the sequence of the exon 2 described here. We did not obtain any positive result for *Hox13* and could not amplify the 3^′^-untranslated regions (UTRs) for *Hox9*, *Hox11*, and *Hox15*. For *Hox11* we obtained the 5^′^-UTR. For *Hox1*, *Hox3*, *Hox4*, *Hox6*, *Hox7*, and *Hox10* we obtained the 3^′^-UTR sequences. For *Hox14* we obtained both 5^′^- and 3^′^-UTRs. For *Hox9* we had only the 5^′^-UTR. We also searched for *Hox13* in the recently published embryonic transcriptome of *B*. *lanceolatum*[39] but did not find any entry. Based on the sequences of the 3^′^-RACE clones, primers were designed to clone part of the 3^′^-UTR. CDS or 3^′^-UTR of each *Hox* gene was cloned into pBluescript SKII+, pCRII-TOPO Dual Promoter vector (Invitrogen), or pGEM-T Easy Vector (Promega), sequenced on both strands and used as templates for ribosynthesis of antisense digoxigenin-labelled probes. All primers used in this study are listed in Additional file 1: Table S1, and the region of the gene used in every case is depicted in Figure 2B. WISH was performed as described [40]. For *Hox3*, *Hox4*, *Hox6*, *Hox7*, *Hox10*, and *Hox14*, only the probes based in the 3^′^-UTR worked in the WISH procedures. None of the probes used gave signals for *Hox2*, *Hox5*, *Hox8*, *Hox9*, *Hox11*, *Hox12*, or *Hox15*. For *Hox1*, the riboprobe used was based on the whole CDS of the gene and the 3^′^-UTR, with both giving the same signal. Sequences of clones used in this study were uploaded to the NCBI GenBank Database under accession numbers JX088059-JX088072 and JX508612-JX508622. After WISH, the embryos were photographed as whole mounts. Several focus planes were merged using Helicon Focus software (d-Studio) for a more accurate identification of expression territories.

### Identification of RAREs

Sequences of lambda phages containing either *B*. *lanceolatum Hox6* and *Hox5* (lambda phage no. λ4131+λ4184 described in [37]) or *Hox14* (lambda phage no. λ4100 in [37]) and their equivalent sequences from *B*. *floridae*[38] were analyzed by nuclear hormone receptor (NHR) scan using default parameter values [41]. We compared the results for sequences in both species and only those elements conserved in both *B*. *floridae* and *B*. *lanceolatum* were considered.

## Results

### *B*. *lanceolatum Hox1*, *Hox3*, and *Hox4* expression patterns: genes that follow colinearity

**Figure 3:**
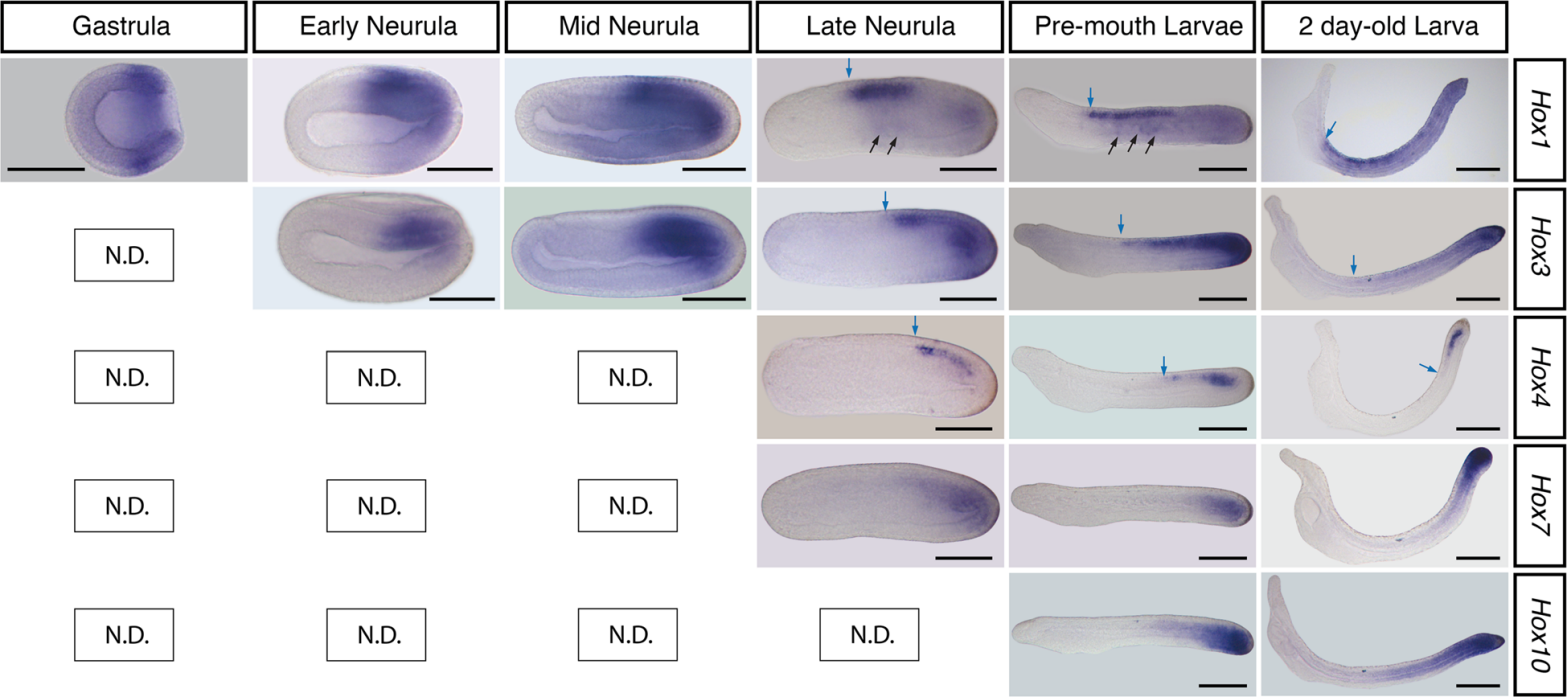
***B. lanceolatum Hox *gene expression patterns.** Expression profile of anterior (*Hox1* and *Hox3*), central (*Hox4* and *Hox7*), and posterior (*Hox10*) genes of the *B*. *lanceolatum Hox* cluster are shown in lateral views of amphioxus embryos from the gastrula stage (left-most panel) to the larval stage (48 h post fertilization, right panels). Whereas *Hox1* was expressed in scattered cells in the epidermis (arrows in the *Hox1* panels of the late neurula and pre-mouth larval stage), this was not clear for *Hox3* and was definitely absent for *Hox4*. Blue arrows indicate the anterior limit of expression in the CNS. In all panels, dorsal is on the top and anterior is to the left. ND, non-detected signal. Scale bars = 100 μm.

The only amphioxus *Hox* genes for which the expression has been reported so far are *Hox1* to *Hox4* and *Hox6* of the Floridian species *B*. *floridae*[18,42]. We used the sibling species *B*. *lanceolatum* to investigate the hitherto unknown expression of several *Hox* genes by WISH. As expected [43], we found that the expression patterns of *B*. *lanceolatum Hox1*, *Hox3*, and *Hox4* in the CNS and mesoderm were very similar to those of their orthologous genes in *B*. *floridae*[18,42], in a clear colinear manner (Figure 3). However, while Schubert *et al*. [19] described a *Hox* nested expression of *Hox1*, *Hox3*, and *Hox4* in scattered epidermal cells (likely involved in the patterning of developing sensory neurons) we found subtle differences in *B*. *lanceolatum*. The epidermal domain of *B*. *lanceolatum Hox1* consisted of scattered cells in a mid-domain of the embryo, as described by Schubert *et al*. [19] (Figure 3, black arrows in *Hox1*). The most anterior limit of *B*. *lanceolatum Hox1* in the CNS coincides with the most anterior limit in the epidermis (Figure 3). This epidermal expression of *Hox1* is also clear from a dorsal point of view (see Additional file 2: Figure S1). However, in the case of *Hox3*, the high level of background obscured this pattern, although from a dorsal viewpoint there appeared to be epidermal expression at the late neurula stage (Additional file 2: Figure S1) as in *B*. *floridae*. Surprisingly, we were not able to detect epidermal expression of *Hox4* (Figure 3), which was expressed only in the CNS.

### A different expression pattern found for amphioxus *Hox6* in the CNS: breaking spatial and temporal colinearity

**Figure 4:**
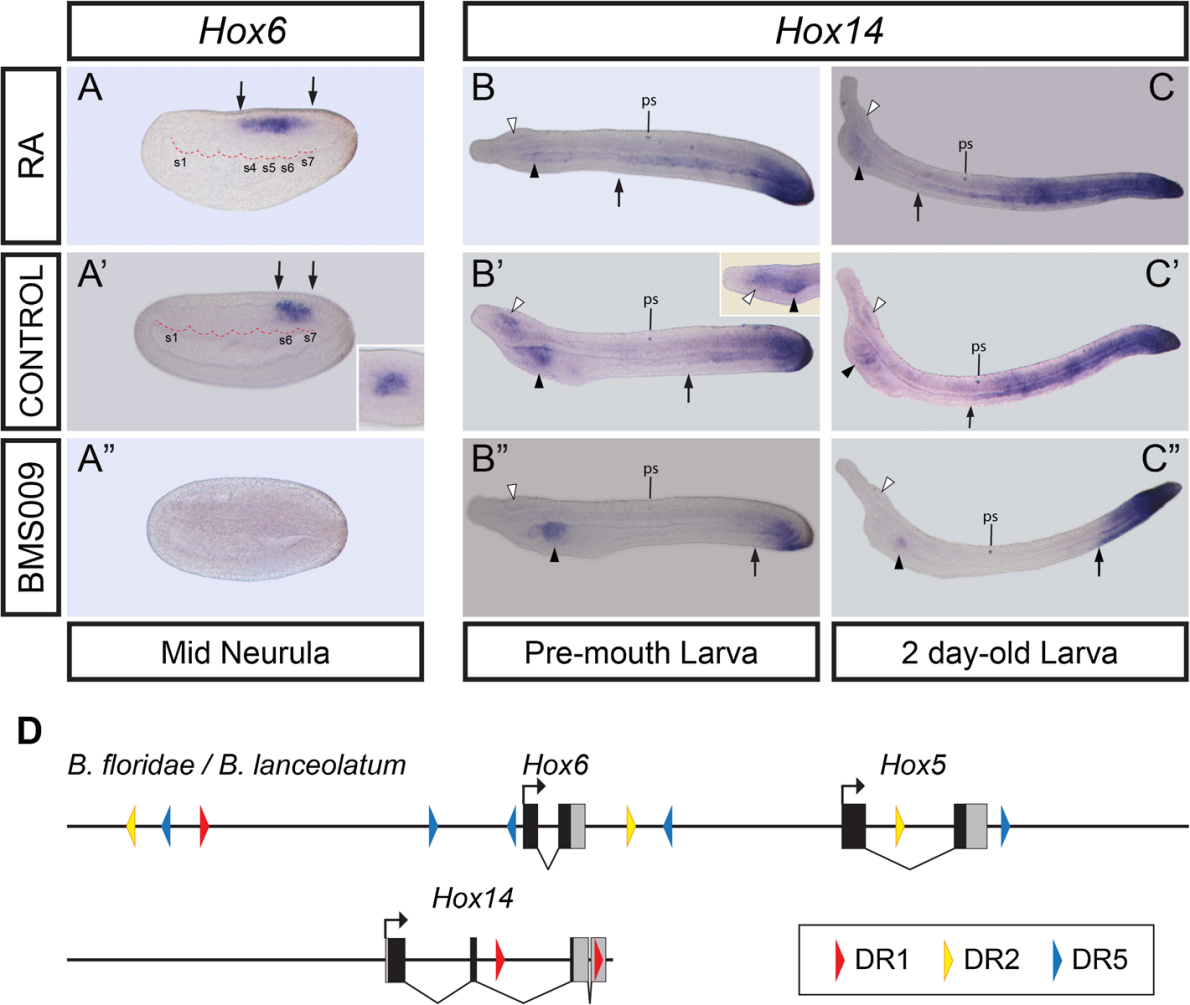
**Retinoic acid(RA)regulation of *Hox6 *and *Hox14*.** Expression patterns of *B*. *lanceolatum Hox6* and *Hox14* and their alterations in embryos treated with RA (upper panels) or the RA antagonist BMS009 (lower panels). (**A**′) *Hox6* was expressed only in a very specific region of the neural plate at the mid-neurula stage and not in the mesoderm (dorsal view in inset). Its anterior limit was enlarged rostrally in RA-treated embryos (**A**) compared with controls (**A**′); the posterior limit remained fixed (limits marked by black arrows). The *Hox6* domain disappeared in BMS009-treated embryos (**A**′′). (**B**′, **C**′) *Hox14* was expressed in the posterior half of the notochord and gut, with a diffuse anterior limit (black arrow),in the cerebral vesicle (white arrowhead) and the left side of the pharyngeal endoderm (black arrowhead). A dorsal view of the head region (inset of **B**′), shows expression in the left part of the pharyngeal endoderm. (**B**, **C**) In RA-treated embryos, *Hox14* expression in the notochord and gut has extended rostrally in both pre-mouth stage and 2-day-old larvae compared with DMSO-treated embryos (**B**′, **C**′), taking the pigment spot (ps) as a reference point. In embryos treated with BMS009, the anterior border of expression was caudally shifted strongly in notochordal and gut domains (**B**′′, **C**′′). Expression in the cerebral vesicle was strongly reduced by RA or RA antagonist treatments (**B** and **C**; **B**′′ and **C**′′,). The pharyngeal domain remained after both treatments (**B**, **C** and **B**′′, **C**′′), even though the severe phenotype in the case of the RA-treated pre-mouth larva showed strongly reduced expression in the pharynx (**B**). (**D**) Schemes of the genomic regions surrounding the loci of *Hox6* and *Hox5* (top), and *Hox14* (bottom) depicting the RAREs of types DR1 (red), DR2 (yellow), and DR5 (blue) that are conserved in both *B*. *floridae* and *B*. *lanceolatum*[37].

Two different expression patterns have been reported so far for *B*. *floridae Hox6*. The first report described *Hox6* as being expressed in all the neural tube posterior to the cerebral vesicle and in the endoderm up to the first gill slit, thereby breaking colinearity [33]. The second report showed a canonical expression in the CNS following colinearity with *Hox14*[18]. To clarify this disparity, we studied the expression of *B*. *lanceolatum Hox6*. Surprisingly, we found a different pattern from those reported above. Unlike *Hox1*, *Hox3*, and *Hox4*, *B*. *lanceolatum Hox6* was expressed in a restricted part of the neural plate at the mid-neurula stage, with very sharp anterior and posterior limits (Figure 4A^′^). The anterior limit was at the level of the intersomitic cleft between somites 5 and 6 and the posterior limit was two somites behind (Figure 4A^′^), unlike *B*. *floridae Hox6*, which Schubert *et al*. described to be expressed from the level between somites 6 and 7 (one somite behind the European amphioxus *Hox6*) rearwards to the tail bud [18]. It is remarkable that *B*. *lanceolatum Hox6* was not expressed in the posterior CNS and tail bud. The anterior limit of *Hox4* in amphioxus is at the level of the middle point of somite 6, half a somite behind *Hox6*, which means that *Hox6* breaks spatial colinearity slightly. Regarding the timing, we have detected *Hox6* exclusively at the mid-neurula stage (between 18 h and 21 h of development at 17°C), earlier than *Hox4* expression (which is expressed from 24 h onwards, from late neurula stage), thus breaking temporal colinearity. *B*. *lanceolatum Hox6* was not detected any later in development. The role of *Hox6* must be very specific both in time and space in patterning of the CNS.

### European amphioxus *Hox7* and *Hox10* expression patterns

Apart from *Hox4* and *Hox6*, no other *Hox* gene expressions have been reported for the central group in cephalochordates. We detected a very weak expression of *Hox7* in the CNS, mesoderm, and tail bud (Figure 3). Due to its weak expression, we cannot rule out the possibility that *B*. *lanceolatum Hox7* was expressed in other tissues. At the late neurula stage, the anterior limit of *Hox7* was at a level equivalent to that of *Hox4*, but the anterior expression was so blurred that establishing a clear boundary was difficult; thus, we could not assess the colinearity relationship between *Hox4* and *Hox7* at this stage. However, from the pre-mouth stage the expression is more posteriorly restricted than for *Hox4*. On the other hand, it began to be expressed after more anterior *Hox* genes, from the late neurula stage, thus retaining temporal colinearity.

No expression of *Hox* genes from posterior groups has been characterized in cephalochordates so far. Here we investigated the expression patterns of *B*. *lanceolatum Hox10* and *Hox14*. We found that *Hox10* expression followed a similar pattern to that for *Hox7*, with very weak expression in the CNS and mesoderm (Figure 3). Again, the weak and blurred expression of *Hox10* makes difficult to exclude the possibility that is actually expressed in other tissues. As for *Hox4* and *Hox7*, the anterior limit of expression of *Hox10* was very diffuse, and although it seemed to be more rostral than the expressions of *Hox4* and *Hox7*, and thus breaking spatial colinearity, the diffuse anterior limit found makes evaluating the colinearity difficult.

### Non-canonical expression of *Hox14*

The most unexpected expression pattern was that of *Hox14*. *B*. *lanceolatum Hox14* is expressed from the pre-mouth larval stage. As for most abovementioned genes, a probe for the coding sequence produced high background and unspecific signals, so we decided again to clone the 3^′^-UTR. We found that it was split into two exons, with a small intron of 45 bp. Amphioxus *Hox14* was expressed in the mid-hindgut, in the posterior part of the notochord, and in the tail bud (Figure 4B^′^, C^′^). Strikingly, *Hox14* was also detected in anterior structures such as the cerebral vesicle and the left side of the pharyngeal endoderm at the level of the endostyle (Figure 4B^′^ and inset).

### Expression of *Hox2*, *Hox5*, *Hox8*, *Hox9*, *Hox11*, *Hox12*, and *Hox15* detected by RT-PCR

Apart from the genes whose expression patterns we have been able to identify, other *Hox* genes were detected by means of RT-PCR during *B*. *lanceolatum* development. Among these genes are *B*. *lanceolatum Hox2*, *Hox5*, *Hox8*, *Hox9*, *Hox11*, *Hox12*, and *Hox15*. *Hox13* was not identified either by RT-PCR or in the recently published embryonic transcriptome of *B*. *lanceolatum*[39]. We obtained the CDSs for *B*. *lanceolatum Hox2*, *Hox5*, *Hox8*, *Hox9*, *Hox11* (first and second exons but not the third), *Hox12*, and the recently discovered *Hox15*[17,37], and 3^′^-UTRs by using 3^′^-RACE RT–PCR for *B*. *lanceolatum Hox2*, *Hox5*, *Hox8*, and *Hox12*. We also amplified the 5^′^-UTR by means of ^′^-RACE RT–PCR of *Hox2*, *Hox9*, and *Hox11*. Nonetheless, we were not able to obtain any signal by WISH (Figure 2B).

The case of amphioxus *Hox2* is quite similar to that of *Hox6*. So far, two reports about its expression have shown two different expression patterns. The first one [42] reported the breaking of colinearity for *Hox2*, while the second [18] reported a colinear expression with respect to *Hox1* and *Hox3*. As with *Hox6*, we wanted to test which *Hox2* expression pattern could be the correct one using *B*. *lanceolatum*. However, although we performed WISH with different probes based on the sequences of the CDS or the 3′-UTR, we were not able to obtain specific signals (Figure 2B).

We found that the 5′-UTR of *Hox9* had two different versions. One was shorter, with a canonical 5′-UTR next to the start codon, and a second larger one was divided into four exons when aligned against the *B*. *floridae Hox* cluster (the complete sequence of the *B*. *lanceolatum Hox* cluster is still not available): three of them were far upstream from *Hox9*. The first exon was placed approximately 5.4 Kbp downstream of *Hox11*, the second was approximately 9 Kbp downstream of *Hox10*, and the third was approximately 25 Kbp upstream of *Hox9*. The fourth corresponded to the canonical 5′-UTR (Figure 2A).

For *B*. *lanceolatum Hox11*, only the first exon has been annotated [37]. Here, we have extended the previously described genomic sequence up to exon 2 (see Methods), which allowed us to find *Hox11* by 5′-RACE RT-PCR. The 5′-UTR was shorter than expected, which means that the previously automatically annotated exon 1 [37,38] was not correct and the actual exon 1 is shorter. As with the other *Hox* genes, a probe based on the coding sequences of exons 1 and 2 gave no signal in WISH (Figure 2B).

### RA and RA-antagonist treatments alter the expression of amphioxus *Hox6* and *Hox14*

The RA-*Hox* system controls the patterning along the A-P axis during development of chordates (for a review see [1]), and such control has been reported widely for anterior *Hox* genes in amphioxus [18-20,23,24,44]. Because we detected a different expression pattern for the *B*. *lanceolatum Hox6* gene than that reported previously [18] and given that amphioxus *Hox14* has been shown to have a non-canonical expression pattern, we treated embryos with RA, the RA antagonist BMS009, or with DMSO as an inert negative control, and carried out WISH experiments.

In RA-treated embryos, the anterior limit of *Hox6* moved rostrally up to the level between somites 3 and 4 (compare Figure 4A with Figure 4A^′^), whereas the posterior limit was unaltered. When treated with the antagonist, *Hox6* expression disappeared. This can be explained by the anterior limit shifting posteriorly to the extreme of its fixed posterior extent, thus making *Hox6* expression disappear (compare Figure 4A^′^ with Figure 4A^′′^). Then, the level of the anterior limit would be that changed when taking the somites as a reference point, as in other more anterior *Hox* genes [18] demonstrating that the changes in expression were regulated by RA (directly or indirectly) and not because of a general shift of internal structures.

In RA-treated larvae, the anterior limit of *B*. *lanceolatum Hox14* expression (at least in the gut) was shifted anteriorly in a significant manner compared with the control, taking as a reference point the mid pigment spot of the CNS. However, it was not so clear for the expression in the notochord (in Figure 4, compare B^′^ and C^′^ with B and C, respectively). In contrast, when treated with BMS009 the expression of *Hox14* in both the notochord and the gut shifted strongly to the posterior (in Figure 4, compare B^′^ and C^′^ with B^′^^′^ and C^′^^′^, respecively). Surprisingly, expression of *Hox14* in the pharyngeal endoderm did not disappear completely in either RA- or BMS009-treated embryos (black arrowheads in Figure 4). Although formation of the pharynx is strongly reduced in RA-treated larvae, with mouth and gill slits failing to form [23], we detected faint expression in the pharynx of *Hox14* in the pre-mouth-stage larvae (Figure 4B, black arrowhead), while *Hox14* was detected clearly in the case of the 2-day-old RA-treated larvae (Figure 4C, black arrowhead). This suggests that regulation of *Hox14* expression in the endostyle is RA independent. The amphioxus cerebral vesicle is a structure that is also reduced in RA-treated larvae but does not disappear, as has been shown using cerebral vesicle markers [18]. Interestingly, the expression of *Hox14* in the cerebral vesicle was extremely reduced with both RA and BMS009 treatments (Figures 4B, 4B^′′^, 4C and 4C^′′^, white arrowheads), suggesting that the cerebral vesicle expression domain is somehow very sensitive to variations in RA level.

RA regulates the expression of its target genes via heterodimers of RAR/RXR that bind RAREs. These heterodimers can bind DR1-, DR2-, and DR5-type RAREs [25]. Using an NHR scan [41], which has been shown to be effective in the prediction of DR2 and DR5 surrounding amphioxus ParaHox genes [45], we looked for RAREs near to amphioxus *Hox6* and *Hox14* genes, using the same genomic regions analyzed previously for non-conserved regions [37] (see Methods). We also screened the corresponding *B*. *floridae* genomic sequences used in our previous comparative regulatory analysis [37], to exclude predictions that have not been conserved between both amphioxus species, because they are probably not real and functional elements. We found that most of the predicted RAREs were not conserved between both species (see Additional file 1: Tables S2 to S5). Thus, we regarded these as false-positives and they were discarded. Using these criteria, we detected one DR2 and three DR5 elements near to *Hox6*, and two DR1 elements within the *Hox14* locus: one within the second intron and the other located in the second exon of the 3′-UTR (Figure 4D).

## Discussion

### Different expression patterns between *B*. *floridae* and *B*. *lanceolatum*

The expression patterns of *Hox1*, *Hox3*, and *Hox4* in amphioxus epidermal neurons have been reported for *B*. *floridae*. However, we did not detect *Hox4* expression in *B*. *lanceolatum* epidermis [19]. Therefore, our data are not consistent with the hypothesis of a ‘skin brain’ (similar to the diffuse net of neurons in hemichordates) in amphioxus [19,46].

As for *Hox6*, unlike the two patterns described previously in the Floridian amphioxus, we have found *B*. *lanceolatum Hox6* only at the mid-neurula stage in a restricted stretch of the neural plate (Figure 4A^′^). The anterior limit of *B*. *lanceolatum Hox6* is one somite level more rostral than that described for *B*. *floridae* by Schubert and colleagues (between somites 7 and 8, [18]) and much more caudal than the anterior limit found by Cohn [33].

One question arises from past and current data: what can explain such different expression patterns in three different experiments? One possibility is that one of the expression patterns of *B*. *floridae* (in the case of Hox6, most likely that reported by Schubert *et al*. [18] rather than that by Cohn [33], because the signal presented by the former seems more reliable than the faint one of the latter report) and those ones presented here for *B*. *lanceolatum* actually reflect a real species-specific difference. If so, it means that the expression of *B*. *floridae Hox6* in the CNS in a colinear manner with the other *Hox* genes is not conserved in *B*. *lanceolatum* CNS patterning. On the other hand, it is possible that experimental consideration such as probe design may explain the differences. For example, since the nucleotide sequences of the homeobox regions of all central *Hox* genes are highly similar (see Additional file 2: Figure S2), a probe spanning this sequence might cause cross-hybridizations and thus partial miss-assignments of expression patterns. In fact, when we used a probe based in the CDS for most of the genes, we obtained either no signal or unspecific staining of the *B*. *lanceolatum* embryos (red lines in Figure 2B). Therefore, we decided to use 3′-UTR-based probes, which are unable to cross-hybridize with other *Hox* genes. The 3′-RACE RT-PCR using gene specific primers designed in the first exon gave only a single band in both *Hox4* and *Hox6* (see Additional file 2: Figure S3), indicating that alternative splicing does not lie behind the difference and that we were detecting only the expression of *Hox4* and *Hox6* transcripts. However, we cannot conclusively discard the presence of alternative transcripts that could account for the different expression patterns obtained upon the use of different probes.

We believe that a revisit of expression patterns in both *B*. *floridae* and *B*. *lanceolatum* and, essentially, in the Asian species *Branchiostoma belcheri*, will help to elucidate if the discrepancies reported come from truly species-specific differences or have an experimental nature.

### Escape from spatial and temporal colinearity

In *B*. *lanceolatum*, *Hox6* was expressed slightly more rostrally than *Hox4* and thus did not maintain spatial colinearity. It was also expressed in an earlier stage (mid-neurula) than that of the onset of *Hox4* expression (late neurula), therefore, *Hox6* also deviated from temporal colinearity. The function of *Hox6* in amphioxus is not known, but it is likely involved in the patterning and regionalization of the CNS in a very specific domain of the neural plate at a very specific time in development. In vertebrates, *Hox6* is expressed in the spinal cord behind the rhombencephalon to the caudal end of the spinal cord and also in the mesoderm. Therefore, the expression of *Hox6* of amphioxus and vertebrates is not conserved. Given that the vertebrate *Hox6* genes maintain both spatial and temporal colinearity, we believe that they represent the ancestral condition, while the expression of amphioxus *Hox6* is probably more divergent. In addition, we have shown that *Hox6* is still regulated (directly or indirectly) by RA, as are the more anterior *Hox* genes [18], suggesting that it is derived from the ancestral state of canonical nested expression together with its mode of regulation.

The expression of amphioxus *Hox6* and the effects of RA and the RA antagonist are very similar to those of the amphioxus ParaHox gene *Gsx*[45]. Amphioxus *Gsx* is expressed in a few cells in the neuroectoderm, at the level of somite 5, just anterior to the *Hox6* domain. Amphioxus *Hox6* and *Gsx* likely participate in the A-P patterning of limited parts of the neuroectoderm in a similar manner, probably in combination with other *Hox* genes that overlap with them. RA treatments enlarge and shift the *Gsx* rostral limit of expression anteriorly whereas RA antagonist treatments make the *Gsx* domain disappear, as in the case of *Hox6*. As with the posterior limit of *Hox6,* which is unaffected by RA treatment, the posterior limit of *Gsx* did not change dramatically with RA treatment. Therefore, as Osborne *et al*. [45] have suggested for *Gsx*, the anterior limit of *Hox6* would be regulated by RA, but the posterior limit would not. Thus, in both *Hox6* and *Gsx*, the loss of the domain following BMS009 treatment can be explained by a caudal shift of the anterior limit until it reaches its posterior one, making the expression to disappear. In vertebrates, it is not known whether *Hox6* paralogs are direct targets of RA regulation. However, other central *Hox* genes such as *HoxA7* and *HoxC8* shift their anterior limit rostrally in the paraxial mesoderm of mouse embryos after RA treatment (*Hox1*.*1* and *Hox3*.*1*, respectively, in [10]), and different *Hox4* paralogs have been shown to be regulated directly by RA [47-49]. However, in other cases such as in the chicken neural tube, the expression levels of genes from *HoxB6* to *HoxB9* have been shown to be refractory to RA treatment [50]. Thus, further investigation is needed in both cephalochordates and vertebrates to understand the ancestral mode of regulation of the central *Hox* genes by RA.

We have also detected the expression of three other Hox genes not studied so far: *Hox7*, *Hox10*, and *Hox14*. While the anterior limit of *Hox7* expression is similar to that of *Hox4* at the late neurula stage, it was more caudal from the pre-mouth larval stage onwards, thus keeping its spatial colinearity. However, the anterior limit of *Hox10* expression in the CNS and mesoderm seemed to be more anterior than that of *Hox4* and *Hox7*. Nonetheless, it is necessary to point out that the anterior limits of amphioxus *Hox7* and *Hox10* were very diffuse, unlike their vertebrate counterparts, which usually display sharp rostral limits, and their colinearity nature is then far from conclusive. Although this difference in the anterior limit between amphioxus and vertebrates might reflect different modes of regulation, we believe that this is the result of the clearly segmental nature of the vertebrate CNS (for example, rhombomeres) compared with the amphioxus CNS. Thus, the real anterior level up to where these genes are expressed in amphioxus might be different to that detected by WISH and thus their colinearity might differ, as discussed here. In vertebrates, *Hox6* and *Hox10* paralogs retain their colinearity and they have important opposite roles: *Hox6* genes encode rib-promoting factors whereas *Hox10* genes are rib-inhibiting [51]. Thus, the colinear expression of *Hox6* and *Hox10* genes in vertebrates is under strong developmental constraints that are not present in amphioxus, allowing these genes to escape from colinearity.

The most striking case of colinearity breakage is that of *Hox14*. Posterior *Hox* genes are involved in the appearance of morphological innovations and are also related to changes in the evolution of the vertebrate bauplan, such as the type of vertebrae [52] or morphological variability within squamates [53]. Interestingly, lamprey and shark *Hox14* genes have non-canonical expression patterns. They are expressed only in the posterior part of the endoderm in the lamprey and in a very specific posteroventral area in the shark surrounding the cloaca [35]. Amphioxus *Hox14* is expressed in anterior structures such as the cerebral vesicle. This is the first *Hox* gene to be detected in such an important organ. In vertebrates, no *Hox* genes are expressed in the midbrain or forebrain and all are excluded from *Otx* and *Pax* expression territories. However, what was thought to be a universal rule is broken in amphioxus. Likewise, the expression of *Hox14* in the pharyngeal endoderm is an exceptional case, because no amphioxus *Hox* gene has been detected earlier in the pharynx. RA regulates the expression of *Hox14* in the notochord and mid-posterior gut in the same manner as anterior genes (enlarged expression anteriorly when embryos are treated with RA, or posterior shift of the anterior limit when treated with an RA antagonist; arrows in Figure 4B-B'', C-C''). On the other hand, *Hox14* seems not to be regulated by RA in the pharynx, because RA treatment did not make the expression disappear, and nor did treatment with the antagonist expand it posteriorly (Figure 4B-B'', C-C'', black arrowheads) as would be expected. Surprisingly, *Hox14* regulation in the cerebral vesicle appeared to be very sensitive to RA, because both excess and a drop in RA level affected its expression strongly (Figure 4B-B'', C-C'', white arrowheads). Thus, the regulatory regions of *Hox14* must be modular. Some expression domains, namely the gut and the notochord, would depend upon RA. Although we cannot discern from the data presented here whether this control is direct or indirect, the presence of DR1 elements within amphioxus *Hox14* locus could give clues for future experiments. Thus, an RA-independent module might regulate the pharyngeal endoderm domain. By contrast, a module sensitive to RA concentration might control the cerebral vesicle domain, perhaps from an indirect effect of RA treatment on some transcription factors that are directly regulated by RA. For vertebrates, a putative regulation of *Hox14* by RA has not been studied. However, other posterior *Hox* genes respond in the opposite way to the anterior genes. For example, *HoxB9* is refractory to RA treatment in the neural tube of chicken embryos, as is *HoxB6*[50], and in the mouse a putative function of RA seems to be to prevent the expression of posterior *HoxD* genes in the anterior domain [54,55].

The uncoupling of vertebrate and amphioxus *Hox14* genes from a canonical *Hox* code is one sign of relaxation of the posterior part of the cluster, but not the only one. Posterior *Hox* genes of cephalochordates, urochordates, echinoderms, and hemichordates do not have clear orthologous relationships to the posterior paralogy groups of vertebrates [34], probably because of the higher evolutionary rate of this class of genes, a phenomenon named deuterostome posterior flexibility [56]. If the posterior *Hox* genes of amphioxus and vertebrates are true orthologs, our data imply that decoupling from the *Hox* code of *Hox14* genes occurred in the last common ancestor of chordates. However, although not conclusively, phylogenetic analyses of deuterostome posterior *Hox* genes, including the recently reported amphioxus *Hox15*, suggest that the posterior *Hox* genes of amphioxus and vertebrates likely originated from independent duplications [17,34,35]. In line with the posterior flexibility hypothesis, the intergenic regions of the posterior *Hox* cluster are less conserved than the anterior ones in both amphioxus [37,38] and gnathostomes [57]. This trend for the posterior *Hox* cluster might be explained if the posterior genes had originated by specific expansions, for example, via tandem duplication, to give 14 *Hox* genes in the last common ancestor of vertebrates and 15 in *Branchiostoma*. Santini *et al*. [57] suggested that this lack of constraint among the posterior *Hox* cluster would have allowed these genes to be involved in the patterning of secondary axes in vertebrates, such as fins and limbs. In amphioxus, the same reasoning would apply to *Hox14* and its unusual expression territories. If the origin of the posterior *Hox* cluster is truly independent, the decoupling of the *Hox14* genes from the classical *Hox* code must have happened independently in the amphioxus and vertebrate lineages.

## Conclusions

The escape of *Hox* genes from canonically nested expression is not unusual. For instance, the *Hox1* and *Hox2* paralogs of vertebrates also do not follow spatial colinearity, so that the anterior limit of *Hox2* is more rostral than that of *Hox1*, which is expressed only in rhombomere 4. This delimited expression of *Hox1* without extension to more caudal regions is similar to that of amphioxus *Hox1* in the CNS. However, it is worth noting that this similarity cannot account for any homology between the *Hox1* domain in amphioxus and rhombomere 4 in vertebrates. Furthermore, in lampreys, temporally colinear expression of *Hox* genes has not been detected [58]. Thus, different *Hox* genes, in different animals escape in one way or another from the colinearity ‘rule’. We believe that these escapes might be associated with the patterning of lineage-specific morphological traits that first requires a loss of the constraint that kept them colinear.

## Competing interests

The authors declare that they have no competing interests.

## Authors’ contributions

JP-A carried out the WISH experiments in amphioxus and performed the genomic DNA analysis for RAREs. NA carried out cloning experiments. SA synthesized and provided the RA antagonist BMS009. SK discussed the results critically. JP-A, SDA, and JG-F conceived the study, participated in the design and coordination of the project, and wrote the draft manuscript. All authors read, discussed, and approved the manuscript.

## Supplementary Material

Additional file 1 **Table S1.** Sequences of primers used in this study to clone B. lanceolatum Hox gene probes. Click here for file

Additional file 2 **Figure S1.** Expression of *B. lanceolatum Hox1* at early neurula (A), mid neurula (B) and late neurula (C) and *Hox3* in the same stages (D, E, F) in dorsal view. The arrows mark the expression in the epidermis, from mid-neurula in the case of *Hox1* and late neurula in *Hox3*. Click here for file
