# Supplementary material for: Broken colinearity of the amphioxus Hox cluster
Source: EvoDevo. 2012 Dec 3;3:28. doi: 10.1186/2041-9139-3-28 (PMC3534614; doi:10.1186/2041-9139-3-28)
Supplement: Additional file 2 — Figure S1. Expression of B. lanceolatum Hox1 at early neurula (A), mid neurula (B) and late neurula (C) and Hox3 in the same stages (D, E, F) in dorsal view. The arrows mark the expression in the epidermis, from mid-neurula in the case of Hox1 and late neurula in Hox3. [file 2041-9139-3-28-S2.pdf]

Additional File 2

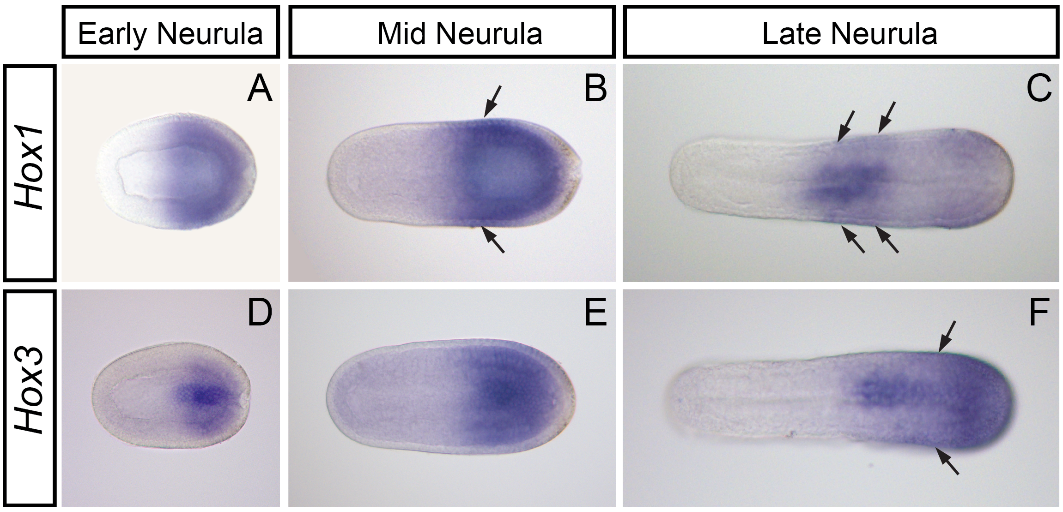

Supplemental Figure S1. Expression of *B. lanceolatum Hox1* at early neurula (**A**), mid neurula (**B**) and late neurula (**C**) and *Hox3* in the same stages (**D-F**) in dorsal view. The arrows mark the expression in the epidermis, from mid-neurula in the case of *Hox1* and late neurula in *Hox3*.

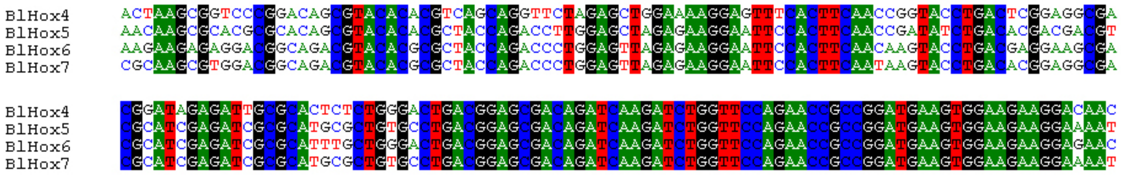

Supplemental Figure S2. Alignment of nucleotide sequences corresponding to the homeobox part of the *B. lanceolatum* (Bl) *Hox4* to *Hox7* genes. The identity between these sequences is extremely high, mainly in the third helix. This fact makes probes based on the homeobox sequence of central *Hox* genes not recommended for *in situ* hybridization experiments.

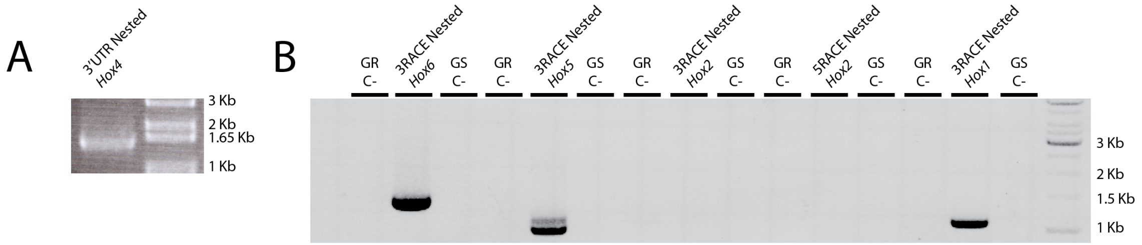

Supplemental Figure S3. (**A**) Nested RT-PCR using pDNR222 cDNA library of *B. lanceolatum* embryos as template for the 3'-UTR of *Hox4* gene. Only a single band was obtained. (**B**) Nested RT-PCR using RACE cDNA of mix embryonic stages of *B. lanceolatum* as template for *Hox1*, *Hox2* (5'- and 3'-UTR), *Hox5* and *Hox6*. Negative controls for the Gene-Racer primer (GR C-) and the Gene-Specific primer (GS C-) for each case is shown. In the case of *Hox2*, we were not able to detect expression using this template, but was later obtained using the pDNR222 cDNA library (data not shown). In case of *Hox5*, we obtained two bands, corresponding to alternative endings of the 3'UTR, but with no alternative splicing (shown in Additional File 2). For both *Hox1* and *Hox6*, only one band was obtained.
